# Prognostic significance of the lymphocyte-to-high-density lipoprotein ratio in long-term efficacy of combined immunotherapy for advanced non-small cell lung cancer

**DOI:** 10.3389/fonc.2025.1712779

**Published:** 2025-11-19

**Authors:** Gaolei Ma, Lijie Ma, Yuan Zhang, Yuanyuan Chen, Yingnan Zhang, Wenwen Guo, Zhiyuan Yao, Guijuan Ji

**Affiliations:** 1 Department of radiotherapy, Xuzhou First People’s Hospital, Xuzhou, Jiangsu, China; 2 Department of Pulmonary and Critical Care Medicine, Affiliated Hospital of Xuzhou Medical University, Xuzhou, Jiangsu, China; 3 First school of Clinical Medical, Gansu University of Chinese Medicine, Lanzhou, Gansu, China; 4 Department of Oncology, Affiliated Hospital of Xuzhou Medical University, Xuzhou, Jiangsu, China

**Keywords:** lymphocyte-to-HDL-C ratio, non-small cell lung cancer, chemo-immunotherapy, prognostic biomarker, nomogram, safety, efficacy

## Abstract

**Background:**

Lung cancer remains the leading cause of cancer incidence and mortality worldwide. Non-small cell lung cancer (NSCLC) accounts for approximately 80%–85% of cases, and the majority of patients are diagnosed at an advanced stage with poor prognosis. Immune checkpoint inhibitors (ICIs) combined with chemotherapy have become the standard first-line treatment for advanced NSCLC, significantly improving survival outcomes. However, considerable inter-individual variability in treatment response persists, underscoring the urgent need for novel predictive biomarkers. Systemic inflammation and immune status are closely associated with immunotherapy efficacy. Lymphocytes play a critical role as effector cells in antitumor immunity, while high-density lipoprotein (HDL), beyond its role in lipid metabolism, also exerts anti-inflammatory and immunomodulatory functions. The lymphocyte-to-HDL ratio (LHR), a composite indicator integrating immune and metabolic status, has demonstrated prognostic value in several malignancies. Nevertheless, its predictive significance in advanced NSCLC patients receiving chemo-immunotherapy remains unclear. This study aims to evaluate the prognostic value of LHR for long-term outcomes in this population, thereby providing insights for individualized treatment strategies.

**Aim:**

To investigate the predictive value of the lymphocyte-to-high-density lipoprotein ratio (LHR) for long-term outcomes in patients with advanced non-small cell lung cancer (NSCLC) receiving chemo-immunotherapy, and to evaluate its potential as a convenient and cost-effective biomarker for guiding individualized clinical treatment.

**Methods:**

This single-center retrospective study included 287 patients with advanced non-small cell lung cancer (NSCLC) who received first-line treatment with immune checkpoint inhibitors (ICIs) combined with platinum-based chemotherapy. Pretreatment lymphocyte-to-high-density lipoprotein ratio (LHR) levels were calculated, and the optimal cutoff value was determined using receiver operating characteristic (ROC) curve analysis. Univariate and multivariate Cox proportional hazards regression analyses were performed to identify independent prognostic factors associated with progression-free survival (PFS) and overall survival (OS). Based on these factors, a nomogram prediction model was developed. Variable selection was guided by clinical relevance, routine applicability, and data availability. Model performance was evaluated using the concordance index (C-index), area under the ROC curve (AUC), and calibration plots.

**Results:**

Based on the optimal cutoff value determined by ROC curve analysis, 287 patients with advanced NSCLC were stratified into a low LHR group (<35.3) and a high LHR group (≥35.3). The median progression-free survival (PFS) was significantly longer in the low LHR group compared with the high LHR group (17.00 [14.00–22.00] vs. 11.80 [9.80–14.50] months; p = 0.028). Similarly, the median overall survival (OS) was 24.00 (21.00–29.00) months in the low LHR group and 18.00 (16.00–20.00) months in the high LHR group (p < 0.001). The objective response rate (ORR) and disease control rate (DCR) were also higher in the low LHR group than in the high LHR group (ORR: 48.92% vs. 35.81%, p = 0.025; DCR: 87.77% vs. 78.38%, p = 0.035). Multivariate Cox regression analysis identified LHR, PD-L1 expression, distant metastasis, and carcinoembryonic antigen (CEA) as independent prognostic factors for both PFS and OS (all p < 0.05). A nomogram prediction model for PFS and OS was subsequently developed based on these factors. In the training cohort, the C-index of the PFS model was 0.73 (95% CI: 0.69–0.78), with an internal validation C-index of 0.78 (95% CI: 0.71–0.85), indicating good discriminative ability. The AUCs for 6- and 12-month PFS prediction were 0.82 (95% CI: 0.76–0.89) and 0.86 (95% CI: 0.75–0.96) in the training cohort, and 0.87 (95% CI: 0.80–0.93) and 0.89 (95% CI: 0.81–0.97) in the validation cohort, respectively. For OS prediction, the C-index values were 0.80 (95% CI: 0.76–0.84) in the training cohort and 0.82 (95% CI: 0.77–0.86) in the validation cohort. The model demonstrated high accuracy in predicting OS at 12, 18, and 24 months: training cohort AUCs of 0.81 (95% CI: 0.74–0.89), 0.85 (95% CI: 0.74–0.91), and 0.94 (95% CI: 0.90–0.98), and validation cohort AUCs of 0.89 (95% CI: 0.80–0.98), 0.88 (95% CI: 0.80–0.96), and 0.82 (95% CI: 0.71–0.93), respectively. Calibration plots showed strong agreement between predicted and observed outcomes, confirming the model’s robustness and clinical applicability.

**Conclusion:**

This study demonstrated that the lymphocyte-to-high-density lipoprotein ratio (LHR) is an independent predictor of long-term outcomes in patients with advanced NSCLC receiving chemo-immunotherapy. A low LHR was associated with improved progression-free survival, overall survival, and higher objective response and disease control rates. The nomogram model incorporating LHR showed favorable predictive accuracy and clinical applicability.

## Introduction

Lung cancer is the leading cause of cancer-related morbidity and mortality worldwide, with non-small cell lung cancer (NSCLC) accounting for approximately 80%–85% of all cases (1). Most patients are diagnosed at an advanced stage, which is associated with limited treatment efficacy and poor prognosis (2). Although chemotherapy has long been the standard treatment for advanced NSCLC, its survival benefit is modest and frequently accompanied by substantial toxicity (3), underscoring the need for more effective therapeutic strategies.

The advent of immune checkpoint inhibitors (ICIs) has significantly improved survival outcomes in a subset of patients with advanced NSCLC (4). Nevertheless, considerable heterogeneity exists in treatment response, and reliable predictive tools to identify patients who would benefit most remain lacking (5). Currently, ICI-based chemo-immunotherapy regimens, such as pembrolizumab in combination with platinum-based chemotherapy, are established as the first-line standard of care, with proven survival benefits in large-scale clinical trials (6, 7). However, not all patients experience durable responses, and some develop resistance or disease progression during treatment. Identifying simple and cost-effective biomarkers to predict treatment efficacy is therefore an unmet clinical need.

Traditional biomarkers, including PD-L1 expression and tumor mutational burden (TMB), are widely used but are limited by factors such as sample heterogeneity, high cost, and inconsistent predictive reliability (8). Recently, immune-nutritional inflammatory biomarkers such as the neutrophil-to-lymphocyte ratio (NLR) and platelet-to-lymphocyte ratio (PLR) have gained attention for their prognostic value in cancer. However, these markers primarily reflect immune status and do not account for metabolic factors. Increasing evidence indicates that tumor progression is not only determined by intrinsic tumor biology but also by host-related factors, including systemic immune and metabolic status (9, 10). In this context, inflammation plays a pivotal role in antitumor immunity (11). Lymphocytes serve as crucial effector cells of the immune response (12), whereas high-density lipoprotein cholesterol (HDL-C), beyond its role in lipid metabolism, also exerts anti-inflammatory and immunomodulatory functions (13, 14).

The lymphocyte-to-HDL-C ratio (LHR) integrates immune function and metabolic status into a single biomarker and has demonstrated prognostic value in several malignancies, including gastric cancer, hepatocellular carcinoma, and colorectal cancer (15, 16). However, its predictive role in advanced NSCLC patients receiving chemo-immunotherapy remains largely unexplored.

Therefore, this study aimed to evaluate the prognostic significance of LHR in advanced NSCLC patients treated with chemo-immunotherapy, and to develop a nomogram model based on LHR and other prognostic factors. Our findings may provide a simple, cost-effective, and clinically applicable tool to guide individualized treatment and improve patient outcomes.

## Materials and methods

### Study design and patients

This single-center retrospective cohort study included patients with advanced non-small cell lung cancer (NSCLC) who were treated at the Affiliated Hospital of Xuzhou Medical University between January 2022 and December 2024. All patients received first-line therapy with pembrolizumab in combination with chemotherapy.

The inclusion criteria were as follows: (1) age >18 years; (2) pathologically confirmed NSCLC; (3) clinical stage III (unresectable) or stage IV disease at initial diagnosis; (4) receipt of at least two cycles of PD-1 inhibitor combined with chemotherapy; and (5) availability of complete medical records, including blood counts, inflammatory biomarkers, and imaging data during immunotherapy.

Exclusion criteria included: (1) concomitant allergic disorders, hematologic diseases, autoimmune diseases, or immunosuppressive conditions during treatment; (2) Eastern Cooperative Oncology Group performance status (ECOG PS) ≥2; (3) history of fever or infection within 1 month prior to treatment; (4) systemic corticosteroid therapy within 1 month prior to treatment; and (5) presence of known driver mutations, including EGFR, ALK, ROS1, or RET alterations.

A total of 287 patients meeting the above criteria were enrolled in this study. Ethical approval was obtained from the Ethics Committee of the Affiliated Hospital of Xuzhou Medical University (approval No. XYFY2024-KL097-01). As this was a retrospective study, the requirement for informed consent was waived by the committee.

### Definition of LHR

The lymphocyte-to-high-density lipoprotein cholesterol ratio (LHR) was calculated as follows: LHR = lymphocyte count (cells/μL)/HDL-C level (mmol/L).

Fasting peripheral venous blood samples were collected from all patients within one week before treatment initiation. Lymphocyte counts were obtained using a routine hematology analyzer, with all samples processed under standard operating procedures to ensure accuracy. Serum HDL-C concentrations were measured using the conventional bromination method on a Beckman Coulter AU5800 biochemical analyzer, following internationally standardized protocols.

The optimal cutoff value of LHR was determined using receiver operating characteristic (ROC) curve analysis, with overall survival (OS) as the endpoint. The area under the curve (AUC) was 0.74 (95% CI: 0.68–0.80). By maximizing the product of sensitivity (78%) and specificity (66%), the optimal cutoff value of LHR was identified as 35.3.

### Data collection

Data were collected from the hospital electronic medical record system and included clinical characteristics such as age, sex, smoking history, body mass index (BMI), and Eastern Cooperative Oncology Group performance status (ECOG PS). Peripheral blood samples were obtained within one week prior to initiation of antitumor therapy for biomarker assessment, including lymphocyte-to-high-density lipoprotein cholesterol ratio (LHR), carcinoembryonic antigen (CEA), carbohydrate antigen 125 (CA125), and squamous cell carcinoma antigen (SCC). LHR was calculated as the ratio of lymphocyte count to HDL-C level. Additional tumor-related variables included pathological subtype, programmed death-ligand 1 (PD-L1) expression, and the presence of distant metastasis.

### Treatment regimen

All patients received standard first-line therapy consisting of pembrolizumab (200 mg, intravenous infusion) combined with platinum-based chemotherapy, administered every 3 weeks. Based on clinical response, the dosing interval for pembrolizumab could be adjusted to every 6 weeks, and treatment was continued until disease progression or unacceptable toxicity occurred.

For patients with adenocarcinoma, cisplatin (75 mg/m²) or carboplatin (AUC 5) was administered in combination with pemetrexed (500 mg/m²) for 4–6 cycles. For patients with squamous cell carcinoma, carboplatin (AUC 6) was administered with either paclitaxel (200 mg/m²) or nab-paclitaxel (100 mg/m²) for 4 cycles. Following 4–6 cycles of induction therapy, patients received pembrolizumab monotherapy as maintenance treatment every 3 weeks, continued until disease progression or the occurrence of intolerable adverse events.

All patients received standard supportive care during treatment, including anti-infective therapy, antiemetic prophylaxis, and supportive transfusion when necessary, to ensure treatment tolerability.

### Evaluation

Treatment efficacy was assessed from the initiation of first-line chemo-immunotherapy, according to the Response Evaluation Criteria in Solid Tumors (RECIST, version 1.1). Tumor response was evaluated by computed tomography (CT) every 8–12 weeks until disease progression or death. Treatment responses were classified as complete response (CR), partial response (PR), stable disease (SD), or progressive disease (PD). To ensure accuracy and consistency of imaging evaluation, all CT scans were independently reviewed by two radiologists with more than three years of experience in oncologic imaging. In cases of disagreement, a third senior radiologist reviewed the scans, and consensus was reached.

Progression-free survival (PFS) was defined as the time from treatment initiation to disease progression or death from any cause. Overall survival (OS) was defined as the time from treatment initiation to death from any cause. Follow-up was conducted through electronic medical records and telephone contact. The last follow-up was performed in December 2024, and the median follow-up duration was 16 months.

### Statistical analysis

All statistical analyses were performed using R software (version 4.5.1). Two-sided tests were applied, and a p-value <0.05 was considered statistically significant. Continuous variables were expressed as median with interquartile range (IQR) after non-normal distribution was confirmed by the Shapiro–Wilk test, and group comparisons were performed using the Mann–Whitney U test. Categorical variables were summarized as frequencies and percentages, and differences between groups were assessed using the χ² test or Fisher’s exact test as appropriate. Variance inflation factor (VIF) was calculated to assess multicollinearity among the variables included in the multivariate model. A VIF value <10 was considered acceptable, and no significant multicollinearity was found between LHR and its individual components (lymphocytes and HDL-C).

Receiver operating characteristic (ROC) curve analysis was conducted to evaluate the predictive value of LHR, with the area under the curve (AUC) calculated to assess diagnostic performance. The optimal cutoff value for LHR was determined by maximizing sensitivity and specificity, and was used to stratify patients for survival analysis. Progression-free survival (PFS) and overall survival (OS) were estimated using the Kaplan–Meier method, and survival differences between groups were compared with the log-rank test.

Univariate and multivariate Cox proportional hazards regression analyses were performed to identify independent prognostic factors for PFS and OS. Variables with p <0.05 in univariate analysis were included in the multivariate model. A nomogram was constructed based on significant prognostic factors, and model discrimination was assessed using Harrell’s concordance index (C-index). The predictive accuracy at different time points was evaluated by calculating time-dependent AUCs from ROC curves. Internal validation was conducted using bootstrap resampling with 1,000 iterations, and calibration plots were generated to assess the agreement between predicted and observed outcomes.

## Results

### Patient characteristics

**Figure 1:**
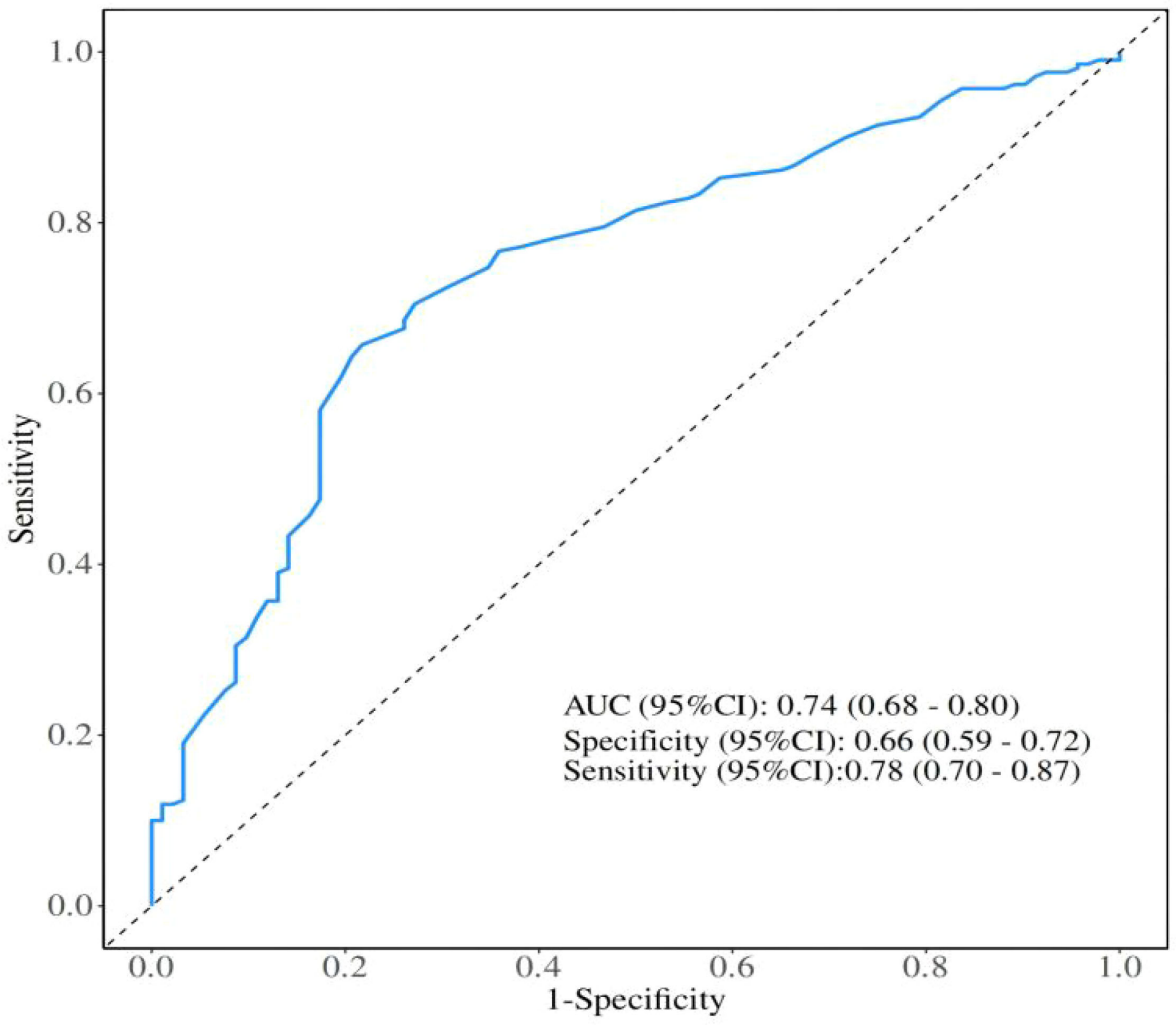
Receiver operating characteristics curve analysis based on Lymphocyte-to-High-Density Lipoprotein Ratio for overall survival. AUC, Area under the curve; CI, Confidence interval.

This study retrospectively analyzed 287 patients with advanced NSCLC who received first-line chemo-immunotherapy at the Affiliated Hospital of Xuzhou Medical University between January 2022 and December 2024. Based on the optimal cutoff value of LHR determined by ROC curve analysis (**Figure 1**), patients were divided into two groups: a low LHR group (LHR < 35.3; n = 139) and a high LHR group (LHR ≥ 35.3; n = 148).

Baseline clinical and tumor characteristics of the study population are summarized in **Table 1**, including sex, age, history of hypertension and diabetes, pathological subtype, PD-L1 expression status, and presence of distant metastasis. Hematological parameters included lymphocyte count, HDL-C level, LHR, carcinoembryonic antigen (CEA), and squamous cell carcinoma antigen (SCCA). Significant differences were observed between the two groups in lymphocyte count, HDL-C level, and LHR (all p < 0.001), while no significant differences were noted for other clinical or tumor-related characteristics.

**Table 1:** Baseline characteristics of Lymphocyte-to-HDL-C Ratio < 35.3 and Lymphocyte-to-HDL-C Ratio ≥ 35.3, *n* (%).

Variables	Overall [n=287]	Low LHR [n=139]	High LHR [n=148]	*P*-value
LHR, M(Q1,Q3)	38.88(28.86,51.93)	28.78(22.28,33.92)	50.40(41.99,62.19)	<0.001
Lym, M(Q1,Q3)	1.50(1.10,1.90)	1.20(1.00,1.50)	1.70(1.40,2.10)	<0.001
HDL, M(Q1,Q3)	1.00(0.83,1.17)	1.10(0.96,1.34)	0.92(0.79,1.04)	<0.001
Sex, n(%)				0.632
Female	87(30.31)	44(31.65)	43(29.05)	
Male	200(69.69)	95(63.35)	105(70.95)	
Age, years				0.479
<60	126(43.90)	64(46.04)	62(41.89)	
≥60	161(56.10)	75(53.96)	86(58.1)	
Smoking history, n(%)				0.618
No	122(42.51)	57(41.01)	65(43.92)	
Yes	165(5749)	82(58.99)	83(56.08)	
Body mass index, kg/m2				0.921
<25	185(64.46)	90(64.75)	95(64.19)	
≥25	102(35.54)	49(35.25)	53(35.81)	
Distant metastasis				0.811
No	128(44.60)	63(45.32)	65(43.92)	
Yes	159(55.40)	76(54.68)	83(56.08)	
ECOG, n(%)				0.334
0	163(56.79)	83(59.71)	80(54.05)	
1	124(43.21)	56(40.29)	68(45.95)	
Histological				0.522
Adenocarcinoma	141(49.13)	71(51.08)	70(47.30)	
Others	146(50.87)	68(48.92)	78(52.70)	
COPD, n(%)				0.965
No	213(74.22)	103(74.10)	110(74.32)	
Yes	74(25.78)	36(25.90)	38(25.63)	
Hypertension, n(%)				0.287
No	200(69.69)	97(62.78)	103(69.59)	
Yes	87(30.31)	42(30.22)	45(30.41)	
Diabetes, n(%)				0.287
No	252(87.80)	125(89.93)	127(85.81)	
Yes	35(12.20)	14(10.07)	21(14.19)	
CEA, ng/mL				0.693
<3	195(6794)	96(62.06)	99(66.89)	
≥3	92(32.06)	43(30.94)	49(33.11)	
CA125, U/mL				0.919
<35	164(57.14)	79(56.83)	85(57.43)	
≥35	123(4286)	60(43.17)	63(42.57)	
SCCA, ng/mL				0.542
<1.5	88(30.66)	45(32.37)	43(29.15)	
≥1.5	199(69.34)	94(67.63)	105(70.95)	
PD-L1 expression				0.300
TPS <50%	135(47.04)	61(43.88)	74(50.00)	
TPS ≥50%	152 (52.96)	78(56.12)	74(50.00)	

Continuous variables are expressed as median (quartile) [M (Q1, Q3)], and the Mann-Whitney U test is used for comparison between groups; categorical variables are expressed as number of cases (percentage) [n (%)], and the chi-square test or Fisher’s exact test (when the expected frequency of cells is < 5) is used for comparison between groups.

LHR, Lymphocyte-to-HDL-C Ratio; ECOG, Eastern Cooperative Oncology Group; COPD, Chronic Obstructive Pulmonary Diseases; CEA, Carcinoembryonic-antigen; CA125, Carbohydrate Antigen 125; SCCA, Squamous Cell Carcinoma Antigen PD-L1, Programmed cell death ligand 1; TPS, Tumor cell Proportion Score.

### Tumor response

Among the 287 patients with advanced NSCLC who received first-line chemo-immunotherapy, no complete responses (CR) were observed. In the low LHR group, 68 patients (48.92%) achieved partial response (PR), compared with 53 patients (35.81%) in the high LHR group. Stable disease (SD) was observed in 55 patients (39.57%) in the low LHR group and 63 patients (42.57%) in the high LHR group. In addition, progressive disease (PD) occurred in 16 patients (11.51%) in the low LHR group and 32 patients (21.62%) in the high LHR group.

The objective response rate (ORR) was significantly higher in the low LHR group compared with the high LHR group (48.92% vs. 35.81%; p = 0.025). Similarly, the disease control rate (DCR) was also superior in the low LHR group (87.77% vs. 78.38%; p = 0.035) (**Table 2**).

**Table 2:** Tumor responses of Lymphocyte-to-HDL-C Ratio < 35.3 and Lymphocyte-to-HDL-C Ratio ≥ 35.3, *n* (%).

Variable	Low LHR index (n = 139)	High LHR index (n = 148)	*X^2^*	*P-value*
PD	17 (11.51)	32 (21.62)		
SD	54 (39.57)	63 (42.57)		
PR	68 (48.92)	53 (35.81)		
ORR			5.05	0.025
Yes	68 (48.92)	53 (35.81)		
No	71 (51.08)	95 (64.19)		
DCR			4.47	0.035
Yes	122(87.77)	116 (78.38)		
No	17 (12.23)	32 (21.62)		

Comparisons between groups were performed using the chi-square test (for categorical variables) or the Mann-Whitney U test (for continuous variables).

LHR, Lymphocyte-to-HDL-C Ratio; CR, Complete response; PR, Partial response; SD, Stable disease; PD, Progression disease; ORR, Objective responds rates; DCR, Disease control rates.

### Progression-free survival and overall survival

**Figure 2:**
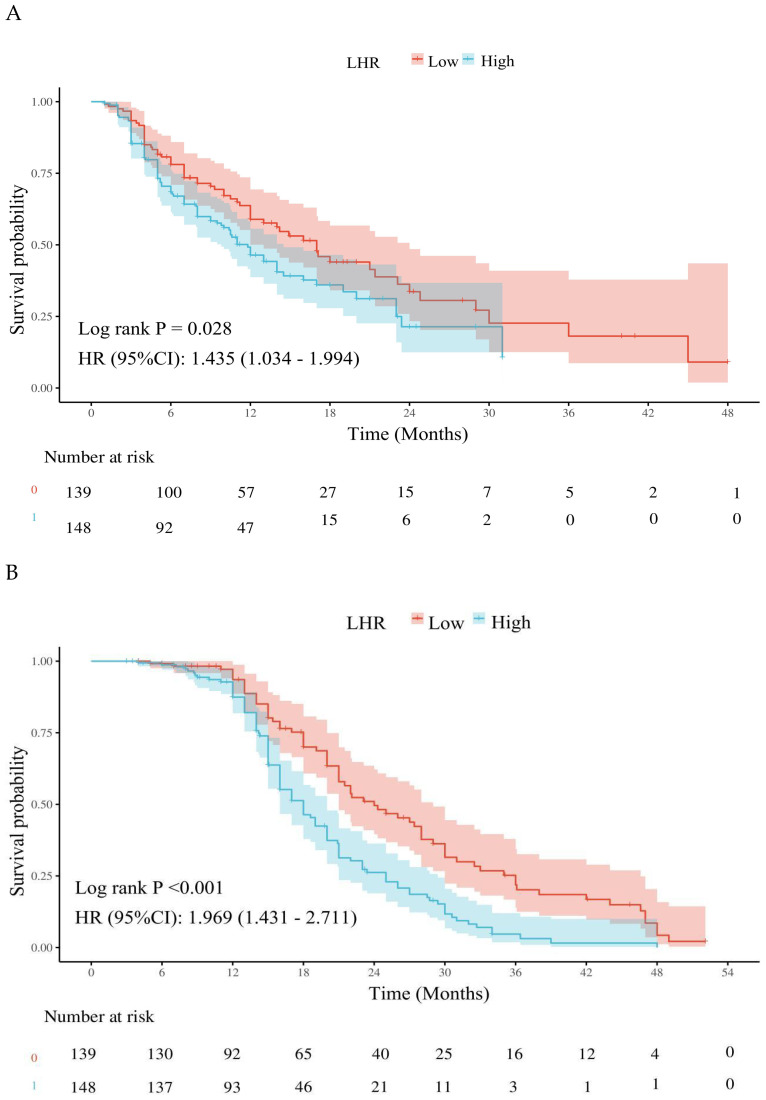
Effects of different lymphocyte-to-high-density lipoprotein ratio on the long-term prognosis of advanced non-Small cell lung cancer patients. **(A)** Kaplan-Meier plot of the LHR < 35.3 and LHR ≥ 35.3 groups; **(B)** Kaplan-Meier plot of overall survival in the LHR < 35.3 and LHR ≥ 35.3 groups. LHR, lymphocyte-to-high-density lipoprotein ratio; HR, Hazard ratio; CI, Confidence interval.

Kaplan–Meier survival analysis demonstrated that patients in the low LHR group had significantly better PFS and OS compared with those in the high LHR group. The median PFS was 17.00 months (95% CI: 14.00–22.00) in the low LHR group versus 11.80 months (95% CI: 9.80–14.50) in the high LHR group (p = 0.028) (**Figure 2A**). Similarly, the median OS was 24.00 months (95% CI: 21.00–29.00) in the low LHR group compared with 18.00 months (95% CI: 16.00–20.00) in the high LHR group (p < 0.001) (**Figure 2B**).

### Cox regression analysis for PFS

To further evaluate the impact of clinical variables on PFS, univariate and multivariate Cox proportional hazards regression analyses were performed (**Table 3**). In univariate analysis, LHR, distant metastasis, carcinoembryonic antigen (CEA) level, and PD-L1 expression were significantly associated with PFS (all p < 0.05). Variables with p < 0.05 in the univariate analysis (LHR, distant metastasis, CEA, PD-L1 expression) were included in the multivariate Cox regression.

**Table 3:** Univariate and multivariate analyses of prognostic factors for progression-free survival.

Factors	Univariate		Multivariate	
	HR (95%*CI*)	*P* value	HR (95%*CI*)	*P* value
Age (<60 vs ≥60), years	1.31 (0.91 - 1.91)	0.536		
Sex (male vs female)	1.14 (0.76 - 1.71)	0.536		
ECOG (0 vs 1)	1.16 (0.80 – 1.69)	0.435		
Histological (others vs adenocarcinoma)	1.22 (0.85 – 1.77)	0.282		
Smoking (no vs yes)	0.76 (0.52 – 1.09)	0.132		
LHR (low vs high)	1.82 (1.24 – 2.67)	0.002	2.07 (1.41 – 3.04)	< 0.001
Distant metastasis (no vs yes)	2.64 (1.76 – 3.97)	<0.001	2.08(1.34 – 3.23)	0.001
COPD (no vs yes)	1.33 (0.89 – 1.99)	0.162		
BMI (<25 vs ≥25), kg/m2	0.47 (0.29 - 0.76)	0.648		
PD-L1 expression (TPS <50% vs TPS ≥50%)	0.45 (0.31 - 0.61)	<0.001	0.57 (0.39 - 0.73)	< 0.001
CEA (<3 vs ≥3), ng/mL	2.86 (1.91 – 4.24)	<0.001	2.06(1.32 – 3.21)	0.001
CA125(<35 vs ≥35), U/mL	0.92 (0.63 – 1.33)	0.639		
SCCA (<1.5 vs ≥1.5), ng/mL	0.86 (0.58 – 1.26)	0.433		

HR, Hazard ratio; CI, Confidence interval; LHR, Lymphocyte-to-HDL-C Ratio; ECOG, Eastern Cooperative Oncology Group; COPD, Chronic Obstructive Pulmonary Diseases; CEA, Carcinoembryonic-antigen; CA125, Carbohydrate Antigen 125; SCCA, Squamous Cell Carcinoma Antigen PD-L1, Programmed cell death ligand 1; TPS, Tumor cell Proportion Score.

Multivariate analysis identified high LHR (HR = 2.07, 95% CI: 1.41–3.04, p < 0.001), presence of distant metastasis (HR = 2.08, 95% CI: 1.34–3.23, p = 0.001), and elevated CEA level (HR = 2.06, 95% CI: 1.32–3.21, p = 0.001) as independent adverse prognostic factors for PFS. In contrast, high PD-L1 expression was significantly associated with longer PFS (HR = 0.57, 95% CI: 0.39–0.86, p = 0.006).

### Cox regression analysis for OS

In the Cox regression analysis for OS (**Table 4**), univariate analysis demonstrated that LHR, distant metastasis, carcinoembryonic antigen (CEA) level, and PD-L1 expression were all significantly associated with OS (all p < 0.05).

**Table 4:** Univariate and multivariate analyses of prognostic factors for overall survival.

Factors	Univariate		Multivariate	
	HR (95%*CI*)	*P* value	HR (95%*CI*)	*P* value
Age (<60 vs ≥60), years	1.22 (0.83 - 1.77)	0.308		
Sex (male vs female)	1.26 (0.84 - 1.89)	0.256		
ECOG (0 vs 1)	1.07(0.73 – 1.56)	0.435		
Histological (others vs adenocarcinoma)	1.31 (0.90 – 1.90)	0.155		
Smoking (no vs yes)	1.06 (0.73 – 1.53)	0.773		
LHR (low vs high)	3.48 (2.25 – 5.37)	<0.001	3.59 (2.33 – 5.54)	< 0.001
Distant metastasis (no vs yes)	2.68 (1.79 – 4.00)	<0.001	2.40(1.56 – 3.70)	< 0.001
COPD (no vs yes)	1.23 (0.81 – 1.86)	0.329		
BMI (<25 vs ≥25), kg/m2	0.89 (0.59 -1.32)	0.548		
PD-L1 expression (TPS <50% vs TPS ≥50%)	0.26 (0.17 - 0.41)	<0.001	0.36 (0.24 - 0.56)	< 0.001
CEA (<3 vs ≥3), ng/mL	2.78 (1.85 – 4.18)	<0.001	1.89(1.20 – 2.97)	0.006
CA125(<35 vs ≥35), U/mL	0.90 (0.62 – 1.31)	0.575		
SCCA (<1.5 vs ≥1.5), ng/mL	1.25 (0.84 – 1.86)	0.276		

HR, Hazard ratio; CI, Confidence interval; LHR, Lymphocyte-to-HDL-C Ratio; ECOG, Eastern Cooperative Oncology Group; COPD, Chronic Obstructive Pulmonary Diseases; CEA, Carcinoembryonic-antigen; CA125, Carbohydrate Antigen 125; SCCA, Squamous Cell Carcinoma Antigen PD-L1, Programmed cell death ligand 1; TPS, Tumor cell Proportion Score.

Multivariate analysis further confirmed high LHR (HR = 3.59, 95% CI: 2.33–5.54, p < 0.001), presence of distant metastasis (HR = 2.40, 95% CI: 1.56–3.70, p < 0.001), and elevated CEA level (HR = 1.89, 95% CI: 1.20–2.97, p = 0.006) as independent adverse prognostic factors for OS. Conversely, high PD-L1 expression was significantly associated with prolonged OS (HR = 0.36, 95% CI: 0.24–0.56, p < 0.001).

### Nomogram construction

In the multivariate Cox regression analysis, LHR, distant metastasis, CEA level, and PD-L1 expression were identified as independent prognostic factors for both PFS and OS. Based on these variables, a prognostic nomogram was constructed using the “rms” package in R software (version 4.5.1). The nomogram was developed by converting the regression coefficients (β values) derived from the Cox model into corresponding point scores, with higher scores assigned to variables exerting a stronger risk effect. For each patient, the individual scores of all variables were summed to generate a total score, which was then mapped to the estimated survival probabilities at specific time points.

**Figure 3:**
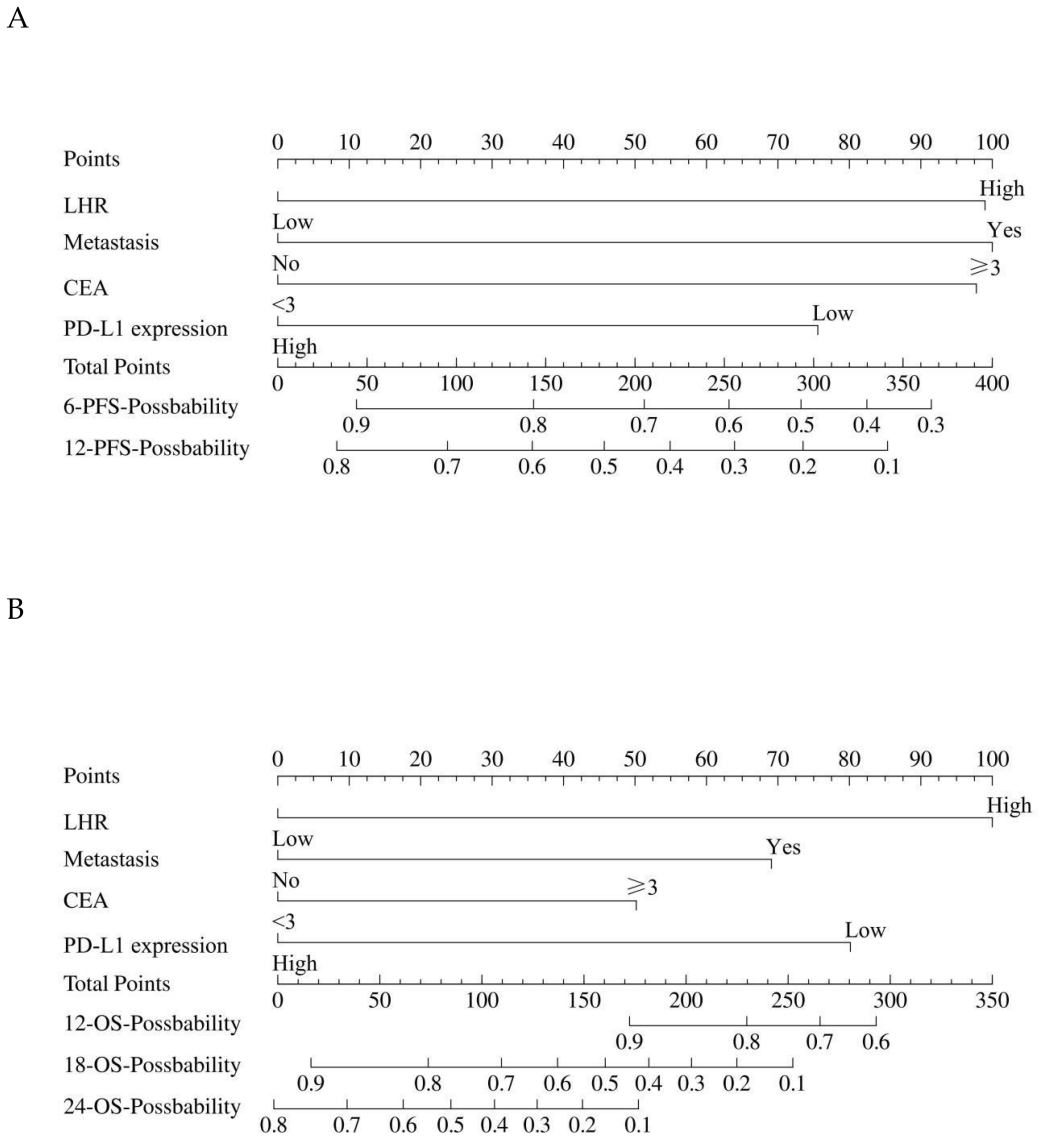
The construction of the nomogram. **(A)** The nomogram for predicting the 6-month and 12-month PFS. **(B)** The nomogram for predicting the 12-month, 18-month, and 24-month OS. The nomogram incorporates key prognostic factors such as LHR, distant metastasis, CEA level, and PD-L1 expression. To calculate the individual score, for each factor, find the corresponding point on the scale. Add the points for each factor to get the total score. This total score is then converted to a probability of PFS and OS using the corresponding line at the bottom of the nomogram. LHR, lymphocyte-to-high-density lipoprotein ratio; CEA, carcinoembryonic antigen; PFS, progression-free survival; OS, overall survival.

Specifically, the PFS nomogram was constructed from these four independent prognostic factors (LHR, distant metastasis, CEA level, and PD-L1 expression) to predict the 6- and 12-month progression-free survival probabilities (**Figure 3A**). Similarly, the OS nomogram was developed using the same set of variables to predict 12-, 18-, and 24-month overall survival probabilities (**Figure 3B**).

### Model validation

In this study, the 287 patients were randomly divided into a training cohort (n = 200) and a validation cohort (n = 87) at a 7:3 ratio (**Table 5**). The nomogram model was constructed based on the training cohort and subsequently validated using internal validation. Internal validation was performed using bootstrap resampling with 1,000 iterations, and model performance was evaluated primarily through the concordance index (C-index), receiver operating characteristic (ROC) curve, and calibration curve.

**Table 5:** Comparison of features between the training and validation sets, *n* (%).

Characteristic	Test (n=87)	Train (n=200)	*X^2^*	*P* value
Age, years			0.22	0.640
<60	40(45.98)	86(43.00)		
≥60	47(54.02)	114(57.00)		
Sex			0.21	0.649
Female	28(32.18)	59(29.50)		
Male	59(67.82)	141(70.50)		
BMI, kg/m^2^			0.31	0.577
<25	54(62.07)	131(65.50)		
≥25	33(37.93)	69(34.50)		
LHR			0.30	0.583
Low	40(45.98)	99(49.50)		
High	47(54.02)	101(50.50)		
ECOG			0.02	0.879
0	50(57.47)	113(56.50)		
1	37(42.53)	87(43.50)		
Smoking			0.07	0.799
No	36(41.38)	86(43.00)		
Yes	51(58.62)	114(57.00)		
Histological			1.20	0.274
Adenocarcinoma	47(54.02)	94(47.00)		
other	40(45.98)	106(53.00)		
Distant metastasis			0.04	0.836
No	38(43.68)	90(45.00)		
Yes	49(56.32)	110(55.00)		
CEA, ng/mL			0.00	0.889
<3	40(45.98)	92(46.00)		
≥3	47(54.02)	108(54.00)		
CA125, U/mL				
<35	51(58.62)	113(56.50)	0.11	0.739
≥35	36(41.38)	87(43.50)		
SCCA, ng/mL				
<1.5	25(28.74)	63(31.50)	0.22	0.641
≥1.5	62(71.26)	137(68.50)		
PD-L1 expression			0.57	0.452
TPS <50%	38(43.68)	97(48.50)		
TPS ≥50%	49(56.32)	103(51.50)		
COPD			0.02	0.997
No	65(74.71)	148(74.00)		
Yes	22(25.29)	52(26.00)		

LHR, Lymphocyte-to-HDL-C Ratio; ECOG, Eastern Cooperative Oncology Group; COPD, Chronic Obstructive Pulmonary Diseases; CEA, Carcinoembryonic-antigen; CA125, Carbohydrate Antigen 125; SCCA, Squamous Cell Carcinoma Antigen PD-L1, Programmed cell death ligand 1; TPS, Tumor cell Proportion Score.

**Figure 4:**
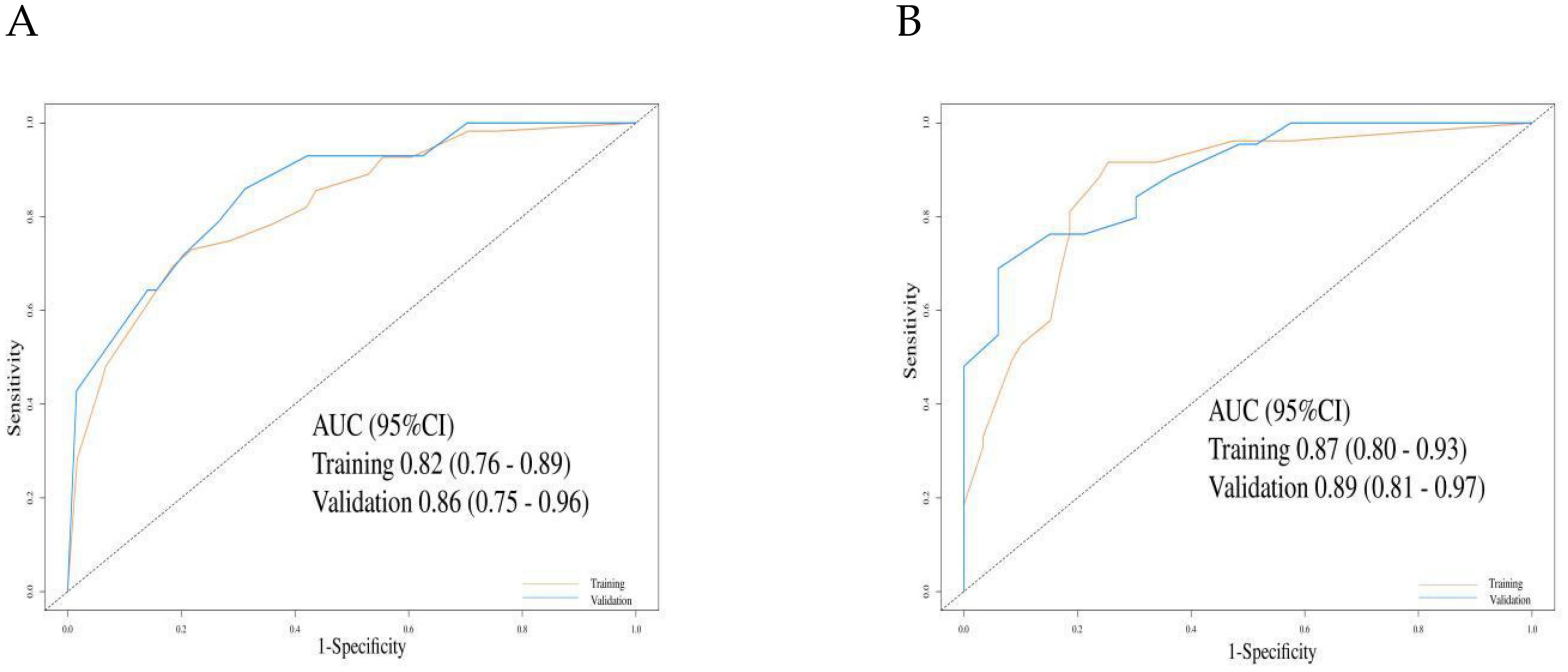
The evaluation of the nomogram for predicting Progression-free survival. **(A)** Graph showing the training set and validation set receiver operating characteristic (ROC) evaluation plots for 6-month prognostic prediction model; **(B)** Graph showing the training set and validation set ROC evaluation plots for 12-month prognostic prediction model. AUC, Area under the curve.

In the training cohort, the C-index for the PFS prediction model was 0.73 (95% CI: 0.69–0.78), and 0.78 (95% CI: 0.71–0.85) in the validation cohort, indicating good discriminative ability. ROC analysis further confirmed the predictive performance of the model: in the training cohort, the AUC for 6-month and 12-month PFS prediction were 0.82 (95% CI: 0.76–0.89) and 0.86 (95% CI: 0.75–0.96), respectively (**Figure 4A**). In the validation cohort, the AUCs for 6-month and 12-month PFS prediction were 0.87 (95% CI: 0.80–0.93) and 0.89 (95% CI: 0.81–0.97), respectively (**Figure 4B**).

**Figure 5:**
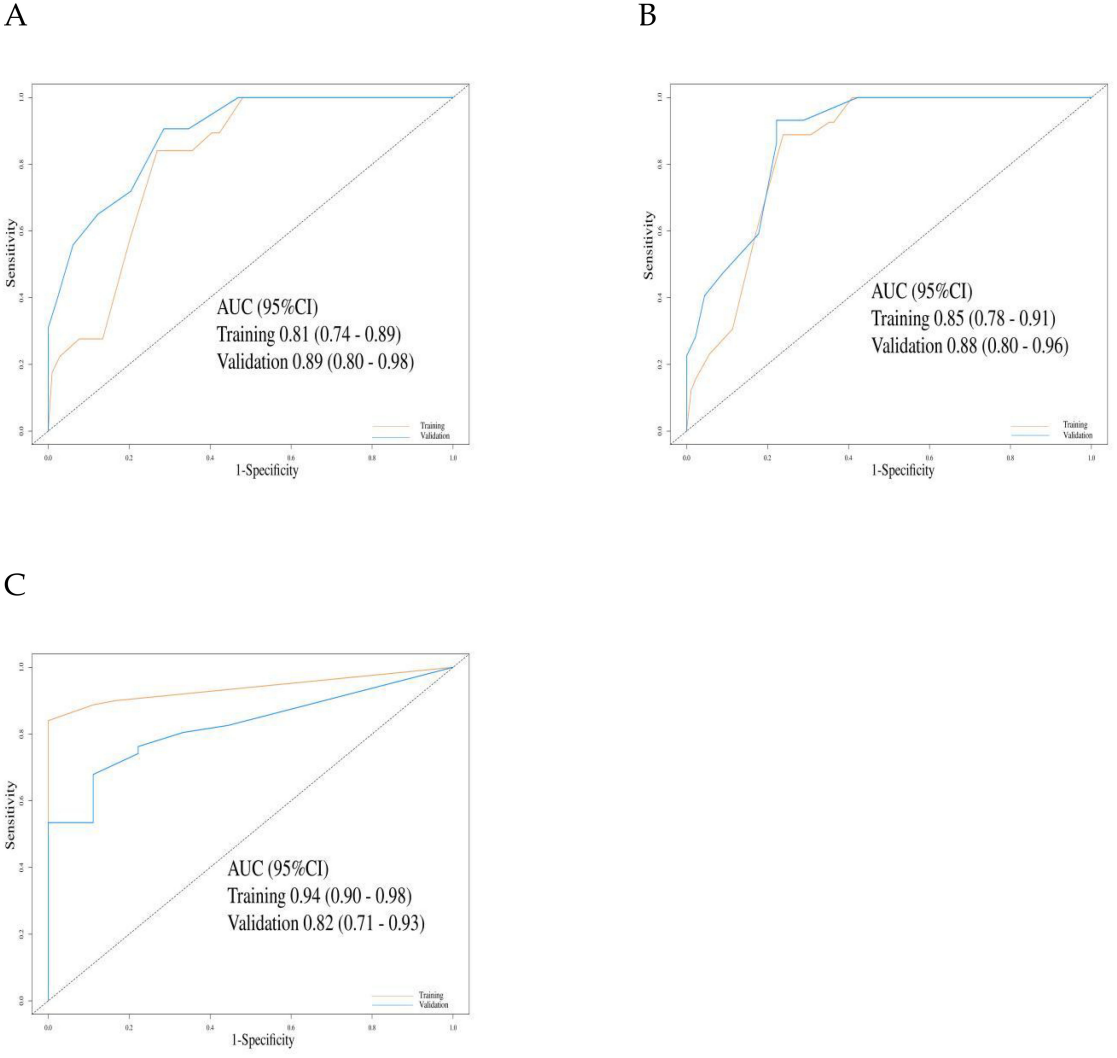
The evaluation of the nomogram for predicting Overall survival. **(A)** Graph showing the training set and validation set receiver operating characteristic (ROC) evaluation plots for 12-month prognostic prediction model; **(B)** Graph showing the training set and validation set ROC evaluation plots for 18-month prognostic prediction model.C: Graph showing the training set and validation set ROC evaluation plots for 24-month prognostic prediction model. AUC, Area under the curve.

For OS prediction, the C-index values in the training and validation cohorts were 0.80 (95% CI: 0.76–0.84) and 0.82 (95% CI: 0.77–0.86), respectively, demonstrating high discriminative ability. The model maintained stable accuracy for predicting OS at 12, 18, and 24 months: in the training cohort, the AUCs were 0.81 (95% CI: 0.74–0.89), 0.85 (95% CI: 0.74–0.91), and 0.94 (95% CI: 0.90–0.98), respectively; in the validation cohort, the AUCs were 0.89 (95% CI: 0.80–0.98), 0.88 (95% CI: 0.80–0.96), and 0.82 (95% CI: 0.71–0.93), respectively (**Figures 5A–C**).

**Figure 6:**
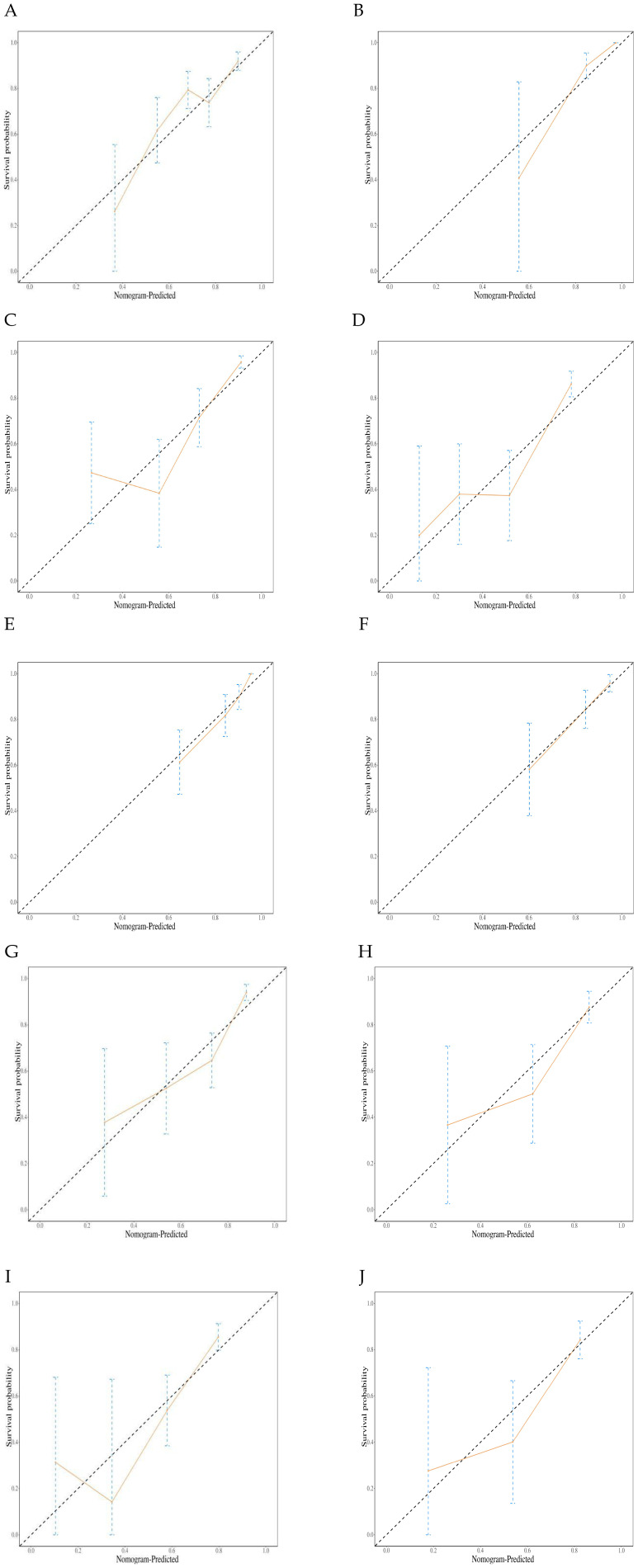
Graph illustrating the calibration plots for a prognostic model. **(A)** Calibration plots for the training set 6-month Progression-free survival (PFS); **(B)** Calibration plots for the validation set 6-month PFS; **(C)** Calibration plots for the training set 12-month PFS; **(D)** Calibration plots for the validation set 12-month PFS; **(E)** Calibration plots for the training set 12-month Overall survival (OS); **(F)** Calibration plots for the validation set 12-month OS. **(G)** Calibration plots for the training set 18-month OS; **(H)** Calibration plots for the validation set 18-month OS. **(I)** Calibration plots for the training set 24-month OS; **(J)** Calibration plots for the validation set 24-month OS.

Calibration curve analysis showed that the predicted survival probabilities were highly consistent with the observed outcomes, indicating that the nomogram model has good predictive accuracy and clinical applicability (**Figures 6A–J**).

### Adverse events

In this study cohort, the majority of patients tolerated chemo-immunotherapy well, with no cases of treatment discontinuation due to adverse events (**Table 6**). The most common adverse events included myelosuppression (23.0%), gastrointestinal reactions (22.7%), rash (16.7%), immune-related pneumonia (12.9%), hepatotoxicity (10.5%), renal and gastrointestinal reactions (4.5%), immune-related pneumonia (3.5%), rash (3.5%), nephrotoxicity (2.8%), hepatotoxicity (2.4%), and hypothyroidism (2.1%). Overall, adverse events were manageable and did not lead to treatment discontinuation. No significant differences were observed between the two groups in the incidence of adverse events (p > 0.05).

**Table 6:** Adverse events associated with pembrolizumab plus chemotherapy in lung cancer patients, *n* (%).

Variables	Total (n = 287)	Low LHR index (n = 139)	High LHR index (n = 148)	Statistic	P
All grades: Myelosuppression, n(%)	66 (23.00)	33 (23.74)	33 (22.30)	χ²=0.08	0.771
All grades: Gastrointestinal response, n(%)	65 (22.65)	31 (22.30)	34 (22.97)	χ²=0.02	0.892
All grades: Hepatotoxicity, n(%)	30 (10.45)	18 (12.95)	12 (8.11)	χ²=1.80	0.180
All grades: Nephrotoxicity, n(%)	32 (11.15)	17 (12.23)	15 (10.14)	χ²=0.32	0.573
All grades: Immune-related pneumonia, n(%)	37 (12.89)	16 (11.51)	21 (14.19)	χ²=0.46	0.499
All grades: Rash, n(%)	48 (16.72)	23 (16.55)	25 (16.89)	χ²=0.01	0.938
All grades: Hypothyroidism, n(%)	25 (8.71)	10 (7.19)	15 (10.14)	χ²=0.78	0.377
All grades: Others, n(%)	40 (13.94)	14 (10.07)	26 (17.57)	χ²=3.36	0.067
≥ 3 grades: Myelosuppression, n(%)	15 (5.23)	7 (5.04)	8 (5.41)	χ²=0.02	0.888
≥ 3 grades: Gastrointestinal response, n(%)	13 (4.53)	6 (4.32)	7 (4.73)	χ²=0.03	0.866
≥ 3 grades: Hepatotoxicity, n(%)	7 (2.44)	5 (3.60)	2 (1.35)	χ²=0.72	0.395
≥ 3 grades: Nephrotoxicity, n(%)	8 (2.79)	5 (3.60)	3 (2.03)	χ²=0.20	0.654
≥ 3 grades: Immune-related pneumonia, n(%)	10 (3.48)	4 (2.88)	6 (4.05)	χ²=0.05	0.825
≥ 3 grades: Rash, n(%)	10 (3.48)	6 (4.32)	4 (2.70)	χ²=0.18	0.672
≥ 3 grades: Hypothyroidism, n(%)	6 (2.09)	1 (0.72)	5 (3.38)	χ²=1.35	0.246
≥ 3 grades: Others, n(%)	11 (3.83)	3 (2.16)	8 (5.41)	χ²=2.05	0.152

The incidence of adverse reactions is expressed as the number of cases (percentage) [n (%)], and the chi-square test is used for comparison between groups.

LHR, Lymphocyte-to-HDL-C Ratio.

## Discussion

This study is the first to validate the independent predictive value of the lymphocyte-to-high-density lipoprotein ratio (LHR) for long-term efficacy in advanced non-small cell lung cancer (NSCLC) patients receiving chemo-immunotherapy. We found that a low LHR was significantly associated with better progression-free survival (PFS) and overall survival (OS), as well as higher objective response rate (ORR) and disease control rate (DCR). Multivariate Cox regression analysis further confirmed that LHR, PD-L1 expression, distant metastasis, and carcinoembryonic antigen (CEA) level were independent prognostic factors for both PFS and OS. Based on these clinical variables, we developed nomogram models for both PFS and OS, which were internally validated to demonstrate good discriminatory power and clinical applicability. These findings provide valuable reference for individualized clinical treatment.

LHR, as a composite marker integrating immune response and metabolic status, reflects the complex interplay between the host immune environment and metabolic health. Lymphocytes, as key effector cells in the body’s antitumor immune response, directly influence the efficacy of immunotherapy (17). Higher lymphocyte counts generally indicate stronger immune surveillance and antitumor activity, which are crucial for the response to immune checkpoint inhibitors (18). At the same time, high-density lipoprotein cholesterol (HDL-C) plays an important role not only in lipid metabolism but also in anti-inflammatory, antioxidant, and immunomodulatory functions (19, 20). HDL-C exerts its effects by inhibiting the secretion of pro-inflammatory cytokines, modulating immune cell function, and reducing systemic inflammation, which indirectly affects the tumor microenvironment (13, 14). Previous studies have shown that low HDL-C levels are associated with higher levels of inflammation and immune escape mechanisms, which may contribute to poor response to immunotherapy (21–23).

Therefore, LHR not only integrates information on immune cell counts and metabolic function but also highlights the role of immune-metabolic imbalance in tumor progression. An elevated LHR reflects a decline in immune cell function and metabolic dysregulation. Immune cells activate key signaling pathways, such as PI3K/Akt, MAPK, and mTOR, through T-cell receptors (TCRs), which directly impact immune response and tumor clearance (24, 25). Meanwhile, HDL-C regulates immune cell membrane lipid composition and promotes anti-inflammatory responses, maintaining immune cell function and enhancing antitumor immunity (26, 27). Lower HDL-C levels weaken T-cell function and may promote immune suppression by tumor-associated macrophages, exacerbating immune escape (28, 29). The elevated LHR, through activation of pro-inflammatory pathways like NF-κB and JNK, further intensifies inflammation in the immune microenvironment, reducing the efficacy of immunotherapy (30–32).

Taken together, LHR serves as a robust indicator that integrates immune response and metabolic dysregulation, providing an effective early warning for tumor immunotherapy responses. The complex interplay between lymphopenia and low HDL-C levels reflects a dynamic immune-metabolic imbalance, which can severely affect tumor progression and response to therapy. This highlights the need for future research to explore the synergistic effect of these two components in greater detail, as well as the potential of LHR to predict therapeutic efficacy and guide personalized treatment strategies.

In recent years, the coupling of immunity and metabolism has become an important research focus in tumor immunology. Several studies have demonstrated that metabolic reprogramming of immune cells directly affects their function, particularly in T-cells and tumor-associated macrophages within the tumor microenvironment (33–35). At the same time, other immune-metabolic-related markers, such as the neutrophil-to-lymphocyte ratio (NLR) and platelet-to-lymphocyte ratio (PLR), have been used for prognosis assessment in various cancers, but their consistency and stability across different tumor types remain variable (36, 37). Compared to these markers, LHR has the advantage of integrating both immune cell quantity and metabolic status, offering a more comprehensive reflection of the immune-metabolic health status of patients. Although LHR has shown good prognostic predictive value in gastric cancer, colorectal cancer, and other malignancies (15), its application in advanced NSCLC treated with chemo-immunotherapy has not been fully explored. Our data indicate that LHR not only reflects the strength of tumor immune response but also serves as an important marker of tumor metabolic reprogramming, overcoming the limitations of traditional biomarkers such as PD-L1 and tumor mutational burden (TMB).

In clinical practice, the detection of LHR can serve as a simple, cost-effective, and easily operable biomarker to predict immune therapy responses in advanced NSCLC patients. The nomogram model based on LHR allows for individualized risk assessment prior to immunotherapy, aiding in patient stratification and treatment decisions. For example, patients with a low LHR typically show better responses to immunotherapy and may be prioritized for chemo-immunotherapy or immunotherapy monotherapy as maintenance treatment; whereas patients with a high LHR may require more intensive monitoring, enhanced supportive care, or alternative treatment strategies such as targeted therapy or chemotherapy. Furthermore, as a hematological marker, LHR is easy to monitor dynamically during treatment, helping to evaluate the effectiveness of immunotherapy and identify the onset of resistance. When combined with other immune therapy biomarkers (such as PD-L1 and TMB), LHR provides a more comprehensive risk assessment, supporting individualized treatment and precision medicine.

Although our study offers valuable insights within a single-center cohort, it is not without limitations. As a retrospective design, it is inherently prone to selection bias and confounding factors, particularly related to the sample selection and data collection process, which may affect the generalizability of the findings. Furthermore, the cutoff value for LHR was determined based on internal ROC curve analysis, and external validation is lacking. Additionally, only the baseline LHR was analyzed, and the prognostic value of its dynamic changes requires further exploration. Future studies should focus on larger, multi-center cohorts to further validate these findings. Second, this study only analyzed baseline LHR and did not investigate how its dynamic changes may affect treatment outcomes. Future research should explore the trend of LHR changes during immunotherapy and assess its potential for real-time monitoring and efficacy evaluation. Third, although we included key clinical variables such as PD-L1 expression, distant metastasis, and CEA, additional immune-metabolic interactions (e.g., immune cell subsets in the tumor microenvironment, metabolic enzymes) were not considered in the model. Incorporating these factors may further improve the accuracy and clinical applicability of the predictive model. In addition, we did not include the chemotherapy regimen in the multivariate analysis. The choice of chemotherapy agents was primarily based on histological subtype (pemetrexed-based regimens for adenocarcinoma and taxane-based regimens for squamous cell carcinoma), which minimized treatment heterogeneity across patients. Furthermore, there was no significant difference in the distribution of chemotherapy regimens between the high and low LHR groups, suggesting that the potential confounding effect of treatment imbalance was limited. However, the exclusion of chemotherapy-related variables remains a limitation of this study, and future large-scale prospective studies are needed to further validate the prognostic stability of LHR across different treatment subgroups. Finally, another limitation of this study is that we only analyzed baseline biomarker data obtained prior to the initiation of treatment, without accounting for the potential influence of dynamic biomarker changes during treatment. Such changes could provide valuable insights into treatment response and long-term prognosis, which warrant further exploration in future studies.

Future research should focus on the following directions: first, conduct multi-center, prospective studies to further validate the predictive performance of LHR in different patient populations; second, combine modern technologies (e.g., immunomics, metabolomics) to explore the mechanistic links between LHR and immunotherapy responses; and third, develop dynamic monitoring tools that combine LHR with other biomarkers to explore its potential as an early warning and intervention tool in immunotherapy. In addition, incorporating post-treatment biomarker evaluations will be essential to enhance our understanding of treatment response and long-term prognosis.

## Conclusion

The lymphocyte-to-high-density lipoprotein ratio (LHR) is an independent predictor of long-term efficacy in advanced NSCLC patients receiving chemo-immunotherapy. A low LHR is associated with improved progression-free survival (PFS), overall survival (OS), and higher response rates. The LHR-based nomogram demonstrated strong predictive accuracy and clinical value. As a simple, cost-effective biomarker, LHR can enhance individualized risk stratification and inform treatment decisions for advanced NSCLC.

## Data Availability

The original contributions presented in the study are included in the article/supplementary material. Further inquiries can be directed to the corresponding authors.
